# Case Report: Unexplained field infertility in an AI purebred Piedmontese bull with normal conventional semen parameters

**DOI:** 10.3389/fvets.2026.1976987

**Published:** 2026-10-06

**Authors:** Irene Viola, Veronica Spalenza, Andrea Albera, Isabella Manenti, Eugenio Martignani, Paola Toschi

**Affiliations:** 1 Department of Veterinary Science, University of Turin, Grugliasco, Italy; 2 Associazione Nazionale Allevatori Bovini di Razza Piemontese (ANABoRaPi), Carrù, Italy

**Keywords:** bull, CASA, flow cytometry, infertility, semen

## Abstract

This case report describes a purebred Piedmontese bull selected for artificial insemination (AI). Despite meeting all conventional semen quality thresholds, the bull showed complete reproductive failure during progeny-testing field inseminations: across all inseminations performed, no pregnancies were confirmed, resulting in a field fertility of 0% and indicating complete infertility under field conditions. To investigate this discrepancy, the bull’s semen was compared with a reference population of 19 Piedmontese bulls managed under identical conditions. Routine ejaculate characteristics, including volume, concentration, and both fresh and post-thaw motility, fell entirely within the expected range, as well as all CASA-derived kinematic parameters. In contrast, flow cytometric evaluation revealed severe functional abnormalities. DNA fragmentation was markedly elevated, and reactive oxygen species (ROS)-related fluorescence intensity was markedly increased, whereas superoxide production was lower than in the control group. Additionally, free thiol content was significantly reduced. Taken together, these alterations indicate not only extensive DNA damage but also a markedly impaired ability of spermatozoa to counteract oxidative stress. These findings demonstrate that profound sperm functional defects may remain undetected through conventional AI-Centre screening, yet critically impair fertility *in vivo*. Incorporating advanced functional assays into routine monitoring may improve early identification of subfertile sires and enhance reproductive efficiency in bovine breeding programs.

## 1 Introduction

Bull fertility is a critical determinant of reproductive efficiency in artificial insemination (AI) programs, where a single sire may produce thousands of semen doses distributed across multiple herds. Under these conditions, even moderate reductions in fertility can accumulate into substantial reproductive and economic losses, affecting conception rates, calving intervals, replacement dynamics, and overall farm profitability (1). For AI centres, undetected subfertility also carries important operational implications, including the use of a higher number of semen doses per pregnancy and the loss of time and resources associated with distributing and testing a bull that may later prove to have compromised semen quality.

Routine semen evaluation in AI facilities relies primarily on conventional parameters such as total and progressive motility and concentration, often supported by Computer-Assisted Sperm Analysis (CASA) to improve objectivity and standardization (2). Although these metrics are essential for quality control and batch acceptance, they remain largely focused on structural and kinematic features. Increasing evidence indicates that such parameters, while necessary, are not always sufficient to predict field fertility. Bulls with comparable motility and morphology may display markedly different conception rates, highlighting a gap between *in vitro* assessments and *in vivo* reproductive performance (3, 4).

This discrepancy has stimulated growing interest in functional sperm biomarkers that capture aspects of cellular physiology not reflected by routine analyses. Parameters such as DNA fragmentation, oxidative status, chromatin compaction, membrane integrity, and mitochondrial function have been associated with fertilizing capacity (5). Flow cytometry offers a high-throughput and objective platform to quantify these markers and supports their potential value in fertility prediction models (6). Nevertheless, functional assays are not yet routinely implemented in commercial semen selection, and their diagnostic contribution in real-world cases remains underexplored.

Here, we describe a bull that exhibited infertility under field conditions despite meeting all conventional semen quality thresholds used for commercial distribution. This case highlights the limitations of standard laboratory assessments and underscores the importance of investigating functional sperm attributes when unexplained field infertility arises. To determine whether underlying biological defects could account for the observed reproductive failure, we performed an extended panel of flow cytometry–based functional analyses and interpreted the results against a structured reference population evaluated under identical analytical conditions.

## 2 Case presentation

### 2.1 Signalment and general Anamnesis

The subject of the present case report was a Piedmontese bull (*Bos taurus*), aged 13 months and weighing approximately 550 kg at the time of evaluation. The animal was housed at the AI station of the Associazione Nazionale Allevatori Razza Bovina Piemontese (ANABoRaPi, Carru’, Italy), under standard management conditions. Feeding management consisted of a ration containing forages and concentrates, formulated to meet maintenance and reproductive requirements, with water provided ad libitum. From a health perspective, the bull was clinically healthy and underwent routine monitoring in accordance with the AI centre’s sanitary and vaccination protocols.

### 2.2 Reproductive history

The bull displayed adequate sexual behavior throughout the collection period, with normal libido and an appropriate mounting response during semen collection. Semen was collected using an artificial vagina according to the standard operating procedures of the officially licensed AI station. The bull was used for semen collection over 5-month period (from May to September 2023), encompassing 18 separate collection days (three successive ejaculate per day) during which ejaculates were regularly evaluated, processed, and frozen in liquid nitrogen in straws following the centre’s internal protocols. For each collection day, semen was assessed both fresh and post-thaw, allowing routine monitoring of ejaculate quality and verification of straw performance after freezing. Field insemination trials [intended for the progeny testing programme, (7)] were conducted in 53 farms, involving 168 females and using a total of 215 straws. Despite the number of inseminations performed, no offspring were confirmed, resulting in a field fertility of 0%. For this reason, the bull semen was not commercialized.

## 3 Methods

To contextualize the subject’s findings, all analyses were performed by comparing the bull’s semen parameters with those of a reference population of 19 purebred Piedmontese bulls (aged 12–15 months) from the same genetic reserve. This comparison group was selected through the same AI-centre selection process and housed under the same management conditions, specifically assembled to represent a range of field fertility outcomes. These 19 reference bulls exhibited a mean field fertility rate (Non-Return Rate) of 71% (range: 54.1–97.3%), which is fully in line with the standard fertility benchmarks for active sires under identical AI centre conditions. Ejaculate quality was further assessed through computer-assisted sperm analysis (CASA) and flow-cytometric evaluation. Specifically, fresh semen analyses and post-thaw CASA evaluations were performed for each production day, while flow-cytometric analyses were conducted post-thaw using three straws per bull.

### 3.1 CASA analysis

Kinematic parameters were assessed using the CASA system (AiStation, SPERM. TECH®, Sperm Analysis Technologies, Valencia, Spain), equipped with a monochrome camera (Basler Ace 2048 × 1,536, 120 fps, mono 2/3″ IMX252, CMOS, global shutter, C-mount, USB3 Vision) mounted on a phase-contrast microscope (Nikon Eclipse E400) at 10 × magnification (8). Frozen semen straws were thawed for 30 s in a 37 °C water bath, diluted 1:2 in Tyrode’s medium, and incubated for 15 min at 37 °C before analysis. A minimum of 500 spermatozoa per sample was evaluated across at least five randomly selected fields. The following motility and kinematic parameters were recorded: total motility (TM, %), progressive motility (PM, %), curvilinear velocity (VCL, μm/s), average path velocity (VAP, μm/s), straight-line velocity (VSL, μm/s), straightness (STR, %), and linearity (LIN, %).

### 3.2 Flow cytometry analysis

Post-thaw semen aliquots were stained with a panel of fluorescent probes targeting specific functional domains (9). Data acquisition settings and gating strategy were established before analysis and applied consistently across all samples. Data were collected using a CytoFLEX flow cytometer (Beckman-Coulter Inc., Brea, CA, USA) equipped with a violet (405 nm), blue (488 nm), and yellow-green (561 nm) lasers and processed with CytExpert software (Beckman Coulter Inc., version 2.4.0.28). Quality control was performed daily as per manufacturer instructions, by running beads (CytoFlex Daily QC Fluorospheres). Unstained semen samples were used to set gates for negative and positive events across all examined parameters. Single stained tubes were prepared for all fluorescent dyes to perform parameter compensation before data acquisition. The general gating strategy involved a first gate on FSC and SSC to broadly select for spermatozoa. The subsequent gate was for Hoechst-33342 positive events to select for nucleated cells. 10,000 Hoechst-33342 positive events were collected for the analysis. The subsequent gate was set to discriminate for vitality: according to the analysis performed, the fluorescent dye used was DRAQ7, SYTOX Green or propidium iodide (PI). A gate for DRAQ7, SYTOX Green or PI negative events was defined, and parameters of interests were then analysed on this subset of events.

One aliquot of spermatozoa was used to assess viability and mitochondrial function using DRAQ7 and JC-1, obtaining Viability (VIT, percentage of live spermatozoa, i.e., DRAQ7-negative events) and High Mitochondrial Potential (HMP, fraction of live cells falling within a gate set for events positive for fluorescent emission at 585 nm).

Oxidative status was evaluated on two other aliquots of spermatozoa: one was stained with SYTOX Green and DHE. DHE-positive, SYTOX Green-negative events provided the percentage of live spermatozoa with high superoxide (O₂^−^·) levels. The other was stained with PI and DCFDA. PI-negative, DCDFA-positive events quantified ROS-related fluorescence intensity within the live population. On another aliquot, DNA integrity was assessed using the SCSA, obtaining the DNA Fragmentation Index (DFI, percentage of sperm with fragmented or denatured DNA) and High DNA Stainability (HDS, proportion of sperm with immature chromatin and abnormally high stainability).

Another spermatozoa aliquot was used to assess chromatin packaging by staining with chromomycin A3 (CMA3). The parameter reported in this study was CMA3_DIFF, calculated as the difference between CMA3-stained and unstained mean fluorescence intensity, used as an indirect indicator of protamine content. For this analysis, a positive control (DTT-treated sample) was also prepared and stained with CMA3. On a final aliquot disulfide bridge status in sperm chromatin was assessed using monobromobimane (mBBr). Two parameters were considered: mBBr_MFI, representing the mean fluorescence intensity of untreated samples and reflecting the abundance of free thiol groups, and mBBr_DIFF, obtained as the difference between DTT-treated and untreated aliquots, used as an indirect measure of the number of disulfide bridges originally present in the samples.

### 3.3 Statistical analysis

Statistical analyses were conducted based on the distributional properties of the control group data. For CASA parameters and the majority of flow cytometry variables that showed a normal distribution according to the Shapiro–Wilk normality test (*p* > 0.05), the deviation of the single-case value from the control distribution was quantified using a standardized *z*-score (*z* = (value−mean)/SD). Alternative statistical approaches were applied to variables that did not meet the assumptions of normality. For mBBr_MFI and DCDFA_MFI, control data were log10-transformed to achieve normality (Shapiro–Wilk test, *p* > 0.05). The single-case value was then compared with controls on the log scale using a modified one-sample *t*-test framework for single-case comparisons, which inherently accounts for the control group’s degrees of freedom (n-1). A standardized *z*-score was also computed on the log-transformed data. Then, HDS and DHE control data were not normally distributed, even after log transformation. Consequently, a nonparametric rank-based single-case comparison was performed. The percentile position of the case score within the control distribution was determined, and statistical significance was calculated using one-tailed exact probability [*p* = 1/(n + 1)]. All analyses were performed using GraphPad Prism 9.

## 4 Results

### 4.1 Fresh ejaculate and post-thaw semen analysis

Ejaculate assessments (*n* = 19 collection days, three ejaculates per day) showed a mean total daily volume of 8.2 mL (range 3.5–11.0 mL) and an average sperm concentration of 1,224 × 10^6^ sperm/mL (range 0.775–1,590 × 10^6^ sperm/mL). Fresh ejaculate motility was consistent across days, with a mean total motility of 70.3% (range: 62–85%) and a progressive motility of 40.2% (range: 36–49%). A total of 9,452 straws were produced. Post-thaw evaluation showed an average of 18.2 × 10^6^ sperm per dose (range 16.4–20.2 × 10^6^). Thawed straw progressive motility averaged 40.3% (range 36–49%) and non-progressive motility averaged 7.4% (range 6–9.2%).

### 4.2 CASA analysis

No differences were observed between the bull and control populations for any CASA-derived parameter (Table 1). All *z*-scores were below ±1.96, indicating that TM, PM, VCL, VAP, VSL, LIN, and STR values fell within the 95% reference interval of the control distribution.

**Table 1:** Comparison between the single-case and control groups across the analysed parameters for CASA and flow cytometry parameters.

Parameter	Control mean ± SD	Range	Single-case value	*z*-score	Statistical significance
TM (%)	66.7 ± 12.0	42.4–84.2	62.0	−0.39	–
PM (%)	61.4 ± 10.1	40.5–75.7	58.20	−0.28	–
VCL (μm/s)	221.0 ± 24.9	182.5–260.8	257.5	1.46	–
VAP (μm/s)	141.7 ± 16.6	107.5–172.8	167.7	1.56	–
VSL (μm/s)	123.8 ± 15.3	93.0–155.0	151.0	1.782	–
LIN (%)	56.1 ± 4.1	48.2–62.5	58.4	0.57	–
STR (%)	85.9 ± 3.0	79.3–91.6	88.4	0.83	–
VIT (%)	44.9 ± 7.7	33.6–59.9	35.9	−1.164	–
HMP (%)	42.4 ± 7.9	30.1–56.3	34.9	−0.94	–
DFI (%)	8.70 ± 2.08	5.34–13.10	22	+6.40	<0.0001
HDS (%)	1.3^*^	0.92–1.58^*^	1.3	57.9%^*^	–
CMA3_DIFF (afu)	247.9 ± 104.1	103.0–483.0	156	−0.88	–
mBBr_MFI (afu)	59,497 ± 3,994	53,817–67,293	49,518	−2.75	<0.0001
mBBr_DIFF (afu)	157,279 ± 13,747	117,870–179,100	162,585,0	0.39	–
DHE (%)	11.59^*^	10.24–13.31^*^	2.38	<1%^*^	0.052
DCFDA_MFI (afu)	1,390 ± 504	862–2,765	16,773	+7.63	<0.0001

Values are reported as mean ± SD for normally distributed data and median [interquartile range] for non-normally distributed data. The asterisk (*) denotes the median; the accompanying range represents the interquartile range and the percentile. *z*-scores were calculated relative to the control distribution. Absolute *z*-scores <1.96 were considered within the 95% reference interval of the control distribution, whereas absolute *z*-scores ≥1.96 and ≥2.58 were considered indicative of values lying outside the 95 and 99% reference intervals, respectively. Then, statistical significance was further assessed only for parameters showing an absolute *z*-score ≥1.96. Units: % (percentage), μm/s (velocity), afu (arbitrary fluorescence units).

### 4.3 Flow cytometry analysis

No substantial deviations from the control distribution were observed for VIT, HMP, CMA3_DIFF, HDS, or mBBr_DIFF, as all values fell within the expected range of variation of the control population (Table 1; Figure 1). DFI showed that the single-case value corresponded to a *z*-score of +6.40, indicating that it was more than six standard deviations above the control mean (*p* < 0.0001). Similarly, the DCDFA_MFI single-case value yielded a *z*-score of +7.63, reflecting an extreme deviation from the control distribution (*p* < 0.0001). In contrast, for DHE, the single-case value (2.38) was lower than all control observations (minimum = 3.68; the exact nonparametric comparison yielded *p* = 0.052). Then, mBBr_MFI showed a marked reduction compared with controls (*z* = −2.25; *p* < 0.0001), indicating a lower abundance of free thiol groups relative to the reference population.

**Figure 1:**
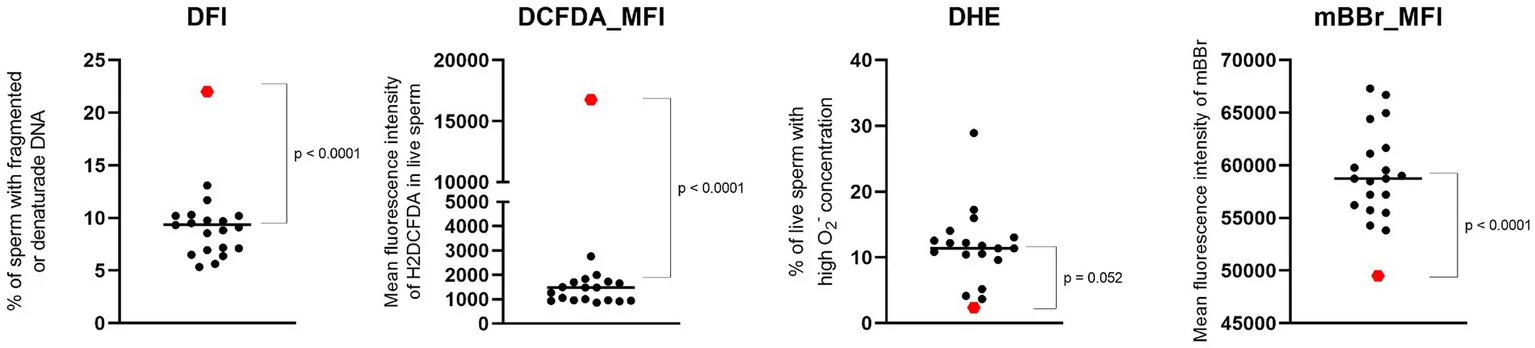
Flow-cytometric parameters showing deviation from the reference population. These visual comparisons highlight the extent to which the bull differs from the population baseline. Scatter plots display individual values for each parameter, illustrating the overall population distribution and the subject’s position relative to this reference. The bull measurements clearly deviate from the expected range, with marked divergence in DNA fragmentation (DFI), total oxidative status (DCDFA_MFI), a borderline reduction in superoxide-related signal (DHE), and decreased free thiols (mBBr_MFI). Each plot reports the magnitude of this deviation relative to the reference population. Statistical significance of differences between the single case and the control group is indicated by the corresponding *p*-value.

## 5 Discussion

This case report presents the situation of a Piedmontese bull whose semen concealed functional alterations that were not evident through routine *in vitro* evaluation. Although conventional CASA parameters met the acceptable thresholds typically used in AI-centre screening, the integration of advanced functional assays revealed biologically relevant abnormalities that were not detectable through routine evaluation and that could provide a coherent explanation for the bull’s infertility performance *in vivo.*

To contextualize these findings, it is important to examine how the bull’s conventional semen parameters compare with those expected for the Piedmontese bulls’ population. Ejaculate volume and sperm concentration were within the normal range reported for bulls routinely introduced at the AI station of ANABoRaPi, as described by Rostellato et al. (10). Likewise, sperm concentration, total motility, and post-thaw progressive motility were fully compliant with the thresholds established by current regulations for bull semen quality (Italian Ministerial Decree No. 403/2000, which governs animal reproduction standards), further confirming that the bull’s infertility was not attributable to deviations in conventional seminal traits.

Once the semen is examined at a deeper functional level using flow cytometry, several biologically relevant deviations emerge, offering a possible interpretation of the bull’s infertility.

First, the bull exhibited a markedly altered functional profile with respect to DNA damage and oxidative stress response. The extremely high DFI may suggest extensive sperm DNA fragmentation, a condition frequently associated with impair fertilization competence (11).

This severe nuclear instability was accompanied by a concomitantly higher DCFDA_MFI and a DHE signal falling below all control observations (*p* = 0.052), a combination potentially consistent with metabolically compromised rather than physiologically low-ROS spermatozoa (12). In particular, the markedly elevated DCFDA_MFI indicates a high level of oxidized probe within the spermatozoa, reflecting an increased overall load of reactive oxygen species (ROS). At the same time, the very low DHE signal (*p* = 0.052) might suggest that superoxide levels are minimal, raising the hypothesis that the oxidative stress observed in this bull is not primarily driven by superoxide but could be instead attributable to other ROS species. Although this specific parameter sits marginally outside the conventional threshold for statistical significance due to the limited control sample size (*n* = 19), this biological reduction aligns with the overall altered oxidative status. To reconcile this apparent divergence, this profile can be biochemically explained by the rapid dismutation of unstable mitochondrial superoxide (O_2_^−^) into more stable downstream species like hydrogen peroxide (H_2_O_2_). This accelerated conversion might prevent superoxide accumulation (depleting the DHE signal) while generating a massive peroxide burden, which could directly account for the extreme DCFDA elevation (*z* = +7.63).

Although this metabolically compromised interpretation remains plausible, future studies should employ specific live-cell fluorophores to confirm this pathway. Specifically, MitoSOX Red could verify if the mitochondria are the primary source of superoxide generation, while C11-BODIPY would determine whether the resulting hydrogen peroxide accumulation ultimately triggers destructive membrane lipid peroxidation.

CASA parameters showed velocity values (VCL, VAP, VSL) in the upper range of the control distribution, though not statistically elevated (with all kinematic *z*-scores remaining within ±1.96) compared to those of the reference bull population. Although these findings could, on their own, be interpreted as indicative of optimal sperm motility, their interpretation might change substantially when integrated with the flow cytometry results. In particular, the extremely high DCFDA_MFI values revealed a condition of marked intracellular oxidative stress. In bovine semen, it could be hypothesized that this phenotype is consistent with a potential early-stage, ROS-induced kinetic overactivation. While true physiological hyperactivation depresses trajectory linearity (LIN and STR) due to asymmetrical beating, this bull maintained normal LIN and STR values. This could indicate a premature, oxidatively driven velocity surge prior to full trajectory destabilization, representing an atypical hyper-velocity state that might reflect underlying cellular dysfunction and typically precedes irreversible cellular damage (13). This interpretation remains strictly speculative and would require direct validation through functional assays, such as capacitation or calcium flux measurements. Nevertheless, while the kinematic profile does not significantly differ from controls, the combination of oxidative stress markers and DNA damage suggests that sperm function may be compromised despite normal motility, consistent with a dissociation between kinematic competence and fertilizing capacity. This hypothesis is further supported by extremely high DNA Fragmentation Index (DFI), indicating that a large proportion of spermatozoa exhibited DNA damage.

Flow cytometry analysis points out another interesting aspect in thiol status balance. Here, we observed that a reduced mBBr_MFI may reflect a depletion of free sulfhydryl groups (-SH). Given that the total amount of disulfide bonds remains unchanged (as shown by mBBr_DIFF), this parameter indicates that the spermatozoa have a severely limited availability of free thiols, which is crucial for protecting against oxidative damage. In sperm cells, these free thiols are predominantly represented by reduced glutathione (GSH), which acts as the main non-enzymatic scavenger against ROS, as reported in bovine species (14). This loss of thiol groups could support the idea that an increased consumption of the endogenous GSH pool, potentially oxidized into glutathione disulfide (GSSG) to counteract the massive oxidative environment (15), as might also be reflected by the high overall intracellular ROS burden. In the context of severe DNA fragmentation, intense oxidative activity, and a tendency of elevated velocity, this apparent collapse of the GSH-mediated antioxidant defense suggests that the cells have lost their redox homeostasis.

In summary, the flow cytometric data indicate that this bull exhibited severe sperm DNA damage, as demonstrated by the extremely high DFI, along with a markedly altered oxidative-stress response, reflected by the combination of a DHE at the absolute minimum of the control, elevated DCDFA_MFI, and reduced mBBr_MFI. Although this remains an isolated case within the AI-selected Piedmontese bull population, it clearly shows that when unexplained field infertility occurs despite adequate routine parameters, targeted in-depth analyses such as functional flow cytometry could be decisive in identifying underlying sperm dysfunction. In this context, while the simultaneous and rapid distribution of semen doses remains critical to maintain short generation intervals in progeny testing schemes, tracking early field data becomes essential. When the first 20–25 field inseminations consistently yield negative results, initiating a targeted functional evaluation of stored straws from that sire could be highly recommended. This approach involves the temporary halt of distribution and the immediate retrieval of a few cryopreserved straws from the AI centre’s bank for external laboratory referral, which would validate suspected subfertility early, enabling timely selection decisions and safeguarding breeding program efficiency.

While this retrospective case was limited to a single time point, future protocols should incorporate longitudinal monitoring concurrently with ongoing field trials to clarify whether these functional abnormalities remain stable or develop progressively over time. Furthermore, this case highlights the potential future value of expanding the post-thawed semen analysis with flow cytometric investigation of DNA fragmentation and oxidative status (16). These analyses, which were long limited to research settings, are now increasingly accessible through routine service testing, with substantially reduced costs (17). Integrating such targeted, second-line functional profiling into progeny testing workflows could facilitate earlier detection of compromised ejaculates when initial fertility outcomes are unfavourable, supporting more informed and effective breeding choices.

## Data Availability

The raw data supporting the conclusions of this article will be made available by the authors, without undue reservation.
